# The brain fatigue syndrome: a cross-diagnostic phenomenon

**DOI:** 10.3389/fnhum.2026.1855514

**Published:** 2026-09-28

**Authors:** Lars Rönnbäck, Paulin Andréll, Helena Filipsson Nyström, Kristina Glise, Anna Grimby-Ekman, Birgitta Johansson

**Affiliations:** 1 Department of Clinical Neuroscience, Institute of Neuroscience and Physiology, Sahlgrenska Academy, Gothenburg, Sweden; 2 Department of Anesthesiology, Intensive Care Medicine and Pain Medicine, Sahlgrenska University Hospital, Gothenburg, Sweden; 3 Department of Anesthesiology and Intensive Care Medicine, Institute of Clinical Sciences, Sahlgrenska Academy, University of Gothenburg, Gothenburg, Sweden; 4 Department of Endocrinology, Sahlgrenska University Hospital, Gothenburg, Sweden; 5 Institute of Medicine, Sahlgrenska Academy, University of Gothenburg, Gothenburg, Sweden; 6 Wallenberg Center for Molecular and Translational Medicine, Gothenburg, Sweden; 7 Gothenburg Centre for Person-centred Care, University of Gothenburg, Gothenburg, Sweden; 8 The Institute of Stress Medicine, Gothenburg, Sweden; 9 School of Public Health and Community Medicine, Institute of Medicine, Sahlgrenska Academy, University of Gothenburg, Gothenburg, Sweden

**Keywords:** acquired brain injury, burnout syndrome’, cross-diagnostic, fatigue, hyperthyroidism

## Abstract

Fatigue arising from diseases or conditions that affect the brain is well recognized. This form of fatigue, brain fatigue, is not comparable to ordinary tiredness that resolves with rest; rather, it reflects a profound and often long-lasting exhaustion. Many individuals struggle in silence, lacking the energy to advocate for their needs. As a result, they may become socially isolated and unable to participate fully in everyday life, despite intact motivation and desire to engage. This fatigue has a substantial impact on work ability, social functioning, and daily activities, making it a serious clinical concern that requires greater attention, improved treatment strategies, and expanded research efforts. In this paper, we refer to this profound fatigue as brain fatigue, typically accompanied by cognitive, emotional, and sensory symptoms. Together, these symptoms may constitute a syndrome, the brain fatigue syndrome (BFS). The aim of this paper is to suggest that BFS, with its cognitive, emotional, and sensory features, appears in a similar way across a wide range of diagnostic groups, despite apparently intact performance on cognitive tests. We propose that BFS appears to represent a manifestation of generalized cerebral involvement, although individuals may concurrently exhibit disease-specific symptoms or deficits related to a brain injury. The symptoms associated with BFS typically persist for the duration of the underlying brain-affecting condition or until recovery occurs. However, in a subset of individuals, BFS may become persistent beyond the expected recovery period, necessitating targeted rehabilitation interventions. We present a theoretical framework for understanding BFS and provide examples of potential treatment strategies for persistent symptoms, as no definitive cure is currently available. In summary, BFS is suggested to reflect brain involvement but is not specific to any single diagnosis.

## Fatigue across diagnoses

In healthcare, clinical attention often centers on symptoms that are specific to a particular disease, which means that fatigue may be overlooked and not fully recognized as a major concern. For many individuals, fatigue subsides as the underlying illness or injury resolves. However, for others, fatigue becomes persistent and chronic, resulting in reduced ability to work or study and limiting participation in social and family life. This form of fatigue reflects an inherent difficulty sustaining activity over time, restricting both the duration, intensity and frequency with which activities can be performed. Endurance is compromised, and this affects many people in society. The type of fatigue discussed here can arise as a consequence of direct or indirect effects on the brain, as seen in acquired brain injuries (Cantor et al., 2013; Johansson et al., 2009; Staub and Bogousslavsky, 2001; Eriksson et al., 2023; Hede Ebbesen et al., 2025; Damsgaard et al., 2014; Veje et al., 2016; Joshi et al., 2023; Day et al., 2016; Bergqvist et al., 2019), neurological disease (Shah, 2009; Friedman et al., 2007; Kluger et al., 2013), endocrine diseases (Johansson et al., 2023; Papakokkinou et al., 2015), systemic inflammatory and autoimmune diseases (Zielinski et al., 2019), myalgic encephalomyelitis/chronic fatigue syndrome (ME/CF) (Hanson, 2023), post-covid (Joli et al., 2022), prolonged stressload (Malmberg Gavelin et al., 2020), neuropsychiatric disabilities (Rogers et al., 2017; Raymaker et al., 2020), psychiatric diseases (Poole-Wright et al., 2025), dementia (Angioni et al., 2023), and chronic nociplastic pain (Koseka et al., 2021).

We propose the term Brain Fatigue Syndrome (BFS) to describe brain-related impairment that emerges as a consequence of injury or disease that affects the brain, either directly or indirectly (Johansson and Rönnbäck, 2025). BFS may occur alongside diagnosis-specific symptoms, and although the initiating mechanisms differ across medical conditions, the underlying brain-related processes contributing to BFS are likely to share common features.

BFS has its origin in long clinical experience, such as in astheno-emotional disorder (Lindqvist and Malmgren, 1993) and post-concussion disorder (King et al., 1995). The cluster of symptoms included in BFS is represented in the assessment scale Mental Fatigue Scale (MFS). The MFS has been evaluated for acquired brain injuries, such as traumatic brain injury (TBI) and stroke (Johansson et al., 2010; Johansson and Rönnbäck, 2014). The MFS has been used for more than a decade, both clinically for a wide range of neurological and acquired brain injuries (Glavå et al., 2025; Johansson et al., 2012; Johansson et al., 2015), and in research involving endocrine groups (Johansson et al., 2023; Papakokkinou et al., 2015; Johansson, 2022) and burnout syndrome (BOS/clinical burnout, Exhaustion Disorder in Sweden, ED) (Johansson, 2022; Skau et al., 2021; van Dam, 2021). The MFS has also been qualitatively evaluated for adults with cerebral palsy (Bergqvist et al., 2019). The MFS captures the exhaustion experienced by the individual, including cognitive, sensory, and emotional symptoms, as well as stress sensitivity and sleep disturbances. All items (see Figure 1) in the MFS show a high correlation with each other (Johansson and Rönnbäck, 2014). Thus, as fatigue increases (MFS total score), cognitive, sensory, and emotional difficulties also increase; and conversely, when fatigue decreases, these symptoms subside. We have also demonstrated this in a treatment study on fatigue after TBI, where the different symptoms covaried in relation to methylphenidate dosage (Johansson et al., 2015).

**Figure 1:**
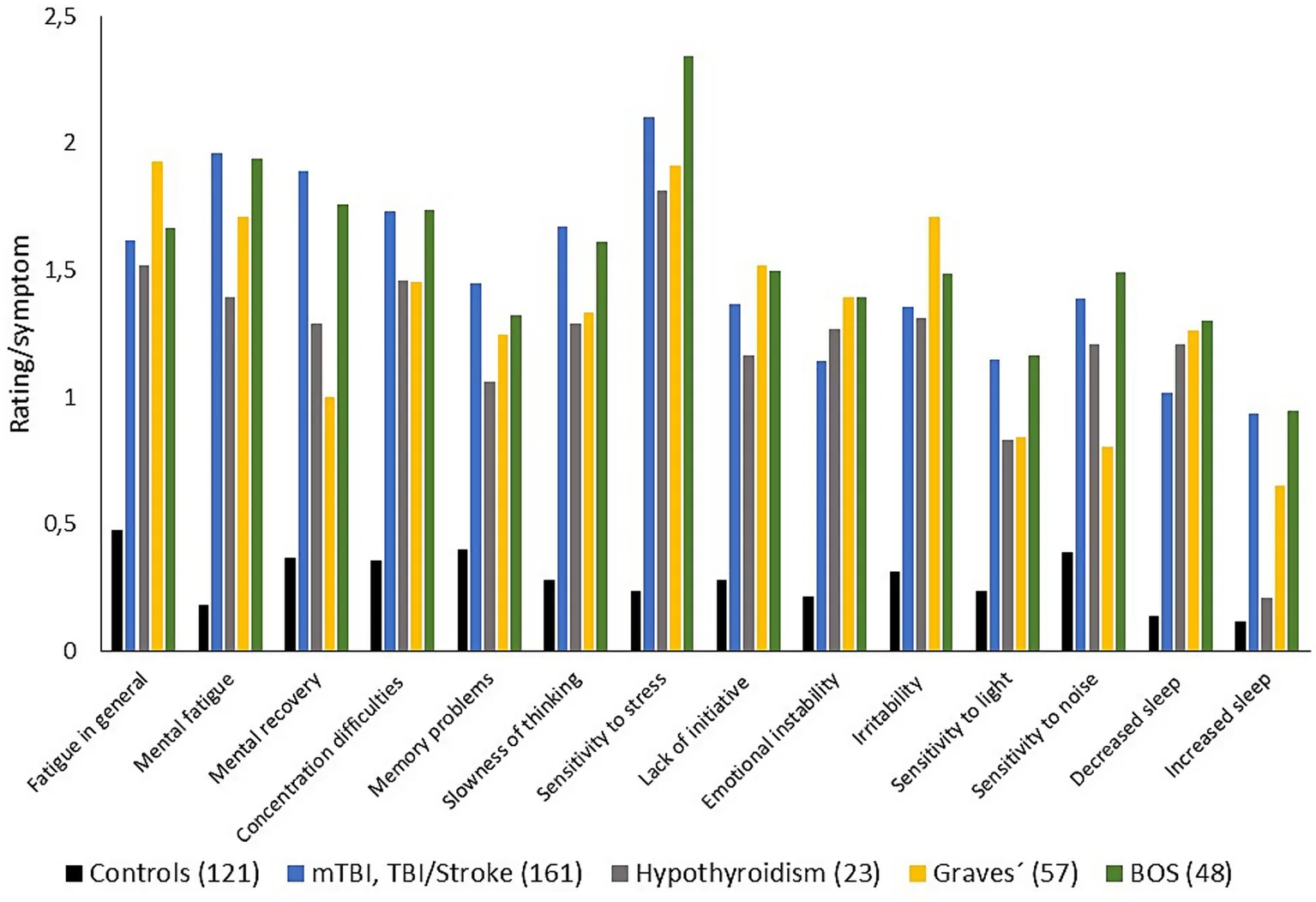
The figure includes several diagnostic groups and presents data only from individuals who score above the cutoff on the Mental Fatigue Scale (MFS) total score, indicating the presence of Brain Fatigue Syndrome (BFS). The figure illustrates how individuals with traumatic brain injury (TBI and mild TBI), stroke, hypothyroidism, Graves’ disease (GD), and burnout syndrome (BOS) report the various symptoms included in the MFS (Y-axis, ratings 0–3 for each symptom). A non-fatigued control group is included for comparison (Johansson et al., 2023; Johansson and Rönnbäck, 2014; Johansson, 2022). Reproduced from [Johansson and Rönnbäck (2025)], licensed under CC BY 4.0.

There is an abundance of fatigue rating scales, more than 70 have been identified (Hjollund et al., 2007). Furthermore, there is still no agreement upon definition of fatigue or how it should be measured. This work needs to be intensified, and there is no consensus. When using the MFS in research across different patient groups, we observe a consistent response pattern for the various MFS items among individuals for whom fatigue is clinically significant, above cutoff at the individual level in comparison with a non-fatigued healthy control group (Johansson and Rönnbäck, 2014) We have found that long-lasting fatigue is common after TBI and even after mild TBI (Johansson et al., 2009), stroke (Johansson and Rönnbäck, 2012) as well as in Graves’ disease (GD) (Johansson et al., 2023). Not everyone is affected, but in BOS most individuals report being fatigued (Johansson, 2022).

Figure 1 presents data exclusively from individuals whose total MFS score exceeds the established cutoff (a total score above 10), indicating clinically significant brain fatigue (Johansson and Rönnbäck, 2014) along with how they responded to the individual items of the MFS. The diagnostic groups included are TBI and mild TBI, stroke (Johansson and Rönnbäck, 2014), hypothyroidism (Johansson, 2022), GD (Johansson et al., 2023), and BOS (Johansson, 2022). The MFS captures the core symptoms that characterize BFS.

In Table 1, the mean values, standard deviations (SD), and number of participants are reported for all individuals included. The table also shows the frequency of those who scored above the cutoff (indicating significant fatigue) in each study. In the study on GD, MFS results are reported both at the time of diagnosis and after 15 months of treatment for hyperthyroidism. There is also a population-based control group, meaning that controls who show significant MFS scores are included as well, representing 14% (Johansson et al., 2023). In a random population-based sample, questionnaires were sent to individuals who had been in contact with primary care and diagnosed with BOS (ED in Sweden), hypothyroidism, or acquired brain injury (TBI and stroke) during the past 5 years. Participants were asked to respond regardless of whether they had problems with brain fatigue, since the purpose was to compare MFS with WAI (Work Ability Index), actual work ability, and to conduct regression analyses. The response rate was 26%. This study did not include a separate control group, and within the diagnostic groups some participants had recovered or had never experienced problems with brain fatigue (Johansson, 2022). The data for TBI and stroke presented in Figure 1 come from the study in which the MFS was specifically evaluated. In this study, individuals with perceived brain fatigue were recruited, along with a control group without brain fatigue (Johansson and Rönnbäck, 2014).

**Table 1:** The table reports statistical data from three studies where the Mental Fatigue Scale (MFS) has been used, and whether data is included in Figure 1 or not.

Study (numbers included)	MFS mean (SD)	% above MFS cutoff	Included in Figure 1	References
Graves’ acute phase (64)	17.5 (6.3)	88	Yes	Johansson et al. (2023)
Graves’ after 15 months (64)	9.5 (6.4)	38	No	Johansson et al. (2023)
Graves’ (63) control population	5.6 (5.5)	14	No	Johansson et al. (2023)
Hypothyroidism (50)	11.5 (7.5)	54	Yes	Johansson (2022)
Burnout syndrome (BOS) (55)	21.0 (5.9)	94	Yes	Johansson (2022)
TBI and stroke (49)	15.4 (7.5)	62	No	Johansson (2022)
TBI and stroke (161)	20.4 (4.7)	100	Yes	Johansson and Rönnbäck (2014)
Controls without brain fatigue (121)	4.3 (0.3)	0	Yes	Johansson and Rönnbäck (2014)

SD, standard deviation.

The different studies therefore have different designs and present observational data. In Figure 1, only participants scoring above the MFS cutoff are included, indicating a significant fatigue based on MFS total score. The purpose is to show how these groups with significant brain fatigue respond to the various symptoms included in the MFS, and to illustrate the similarities in response patterns, suggesting that BFS can be assumed to be very similar across diagnostic groups, even though the prevalence of brain fatigue varies between population-based diagnoses.

We have also shown that work ability is related to MFS, the higher the score, the lower the work capacity, which is a clear indicator that BFS has substantial impact on an individual’s ability to perform work (Johansson, 2022; Palm et al., 2017).

## How to recognize brain fatigue syndrome

The central issue is profound fatigue and the inability to regain energy quickly. This often leads to marked fluctuations during the day, as well as between days and weeks, all depending on what the person does and how much effort the activity requires. In addition, there are distinct cognitive difficulties, including reduced ability to maintain concentration over time with loss of focus and increased distractibility, memory problems, and slowness in thinking. The sensory problems involve noise, and light sensitivity, being experienced as more intense and more intrusive than before the onset of illness. The emotional difficulties include irritability and increased tearfulness. Although these three symptom domains differ in magnitude, they co-occur to varying degrees within individuals and are interrelated (Figure 2).

**Figure 2:**
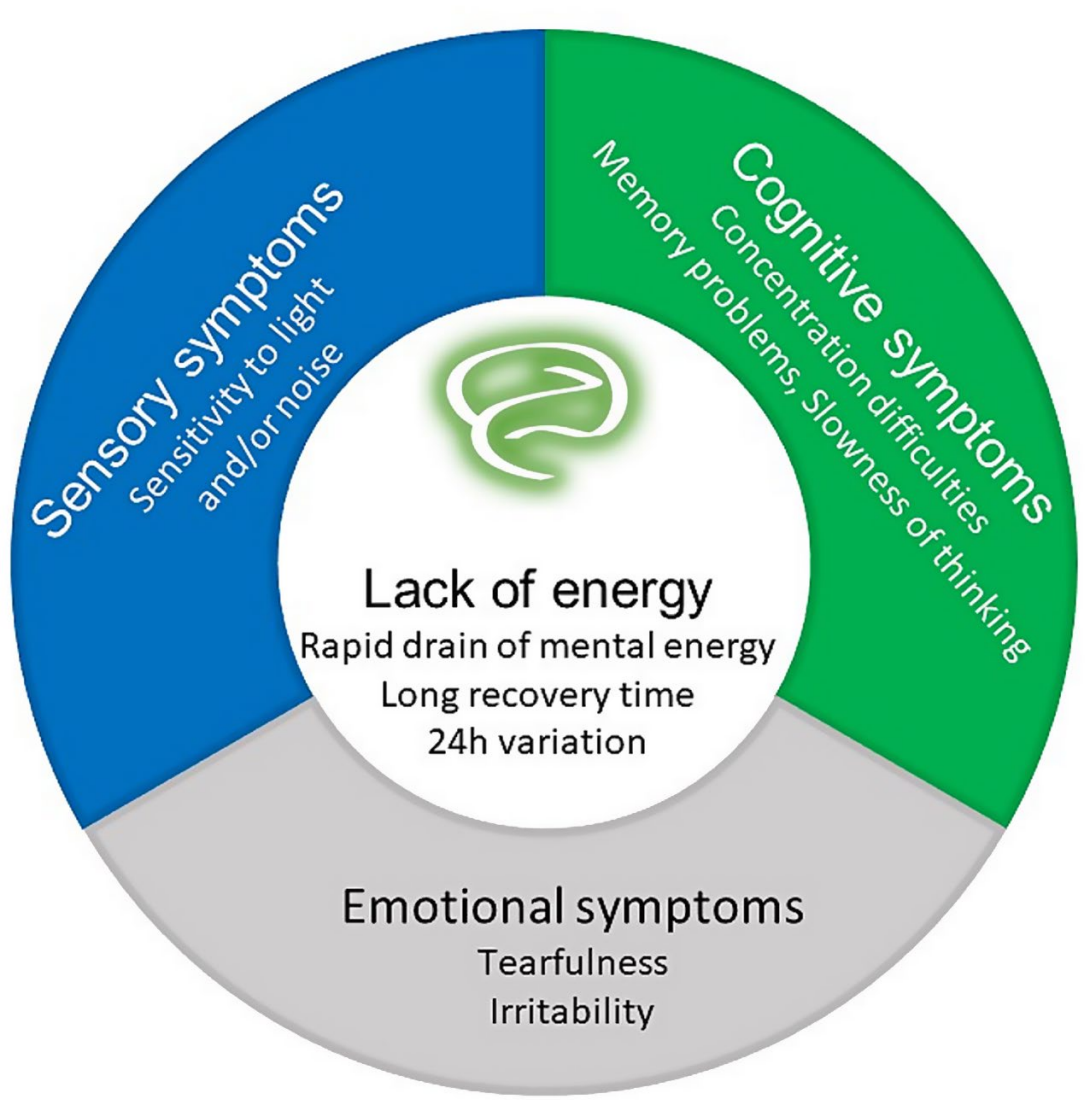
The figure illustrates the symptoms associated with Brain Fatigue Syndrome. The symptoms are grouped into three domains: cognitive, emotional, and sensory. The figure is intended to show the association of symptoms with these domains and does not represent proportions.

All these symptoms are suggested to be related to disturbed brain function generally linked to central neurotransmitters, reduced in efficiency due to inflammatory-reactive astrocytes, according to the hypothesis explained below. Disturbed sleep is also common, where sleep does not provide adequate recovery. Increased headaches often occur in connection with greater mental effort (Johansson et al., 2009; Johansson and Rönnbäck, 2025; Johansson and Rönnbäck, 2014).

## Hypothetical mechanisms underlying BFS

BFS is proposed to arise from an impact on the brain, either directly or indirectly via the blood–brain barrier triggered by a stressor such as disease, injury, or systemic inflammation, including autoimmune processes (Zielinski et al., 2019; Johansson and Rönnbäck, 2025; Tammelin et al., 2026). These stressors can lead to inflammatory activation of astrocytes. Astrocytes may become reactive due to direct brain injury or disease, but also through activation of the brain’s microglial cells. Once astrocytes enter this inflammatory-reactive state, disturbances in glutamate regulation emerge.

Impaired glutamate signaling may provide a unifying explanation for several characteristic features of BFS: insufficient energy supply to the brain, dysfunctional transmission across multiple neurotransmitter systems, and increased nonspecific neuronal activation involving larger and partly irrelevant/nonspecific neural circuits. Together, these processes can lead to reduced glucose (energy) availability and less efficient information processing manifesting as BFS.

All brain cells require energy to sustain their function, although the magnitude and nature of these energy demands differ between cell types. Evolutionarily, both the neocortex and mitochondria have been biochemically optimized for efficient energy transformation (Mosharov et al., 2025). As a result, complex cognitive functions may be especially sensitive to disruptions in energy metabolism. However, a significant knowledge gap remains. While modern neuroimaging techniques enable the large-scale mapping of brain metabolism, a more comprehensive understanding of how these measurements relate to the underlying cellular and molecular mechanisms is still needed. Bridging this gap will be crucial for linking systems-level observations to cell-specific metabolic processes.

### Preclinical data

This hypothesis is grounded in well-established preclinical research demonstrating that neurological and systemic diseases can affect the brain both directly and indirectly, leading to an initial inflammatory response (Galea, 2021). This response may result in astrocyte dysfunction, influencing the performance of the brain’s main support cells (Korn et al., 2007; Hansson and Rönnbäck, 1995; Hertz and Zielke, 2004). In conditions such as TBI, stroke, central nervous system (CNS) inflammation, or chronic stress, inflammatory response can cause astrocytes to become reactive and less efficient at handling glutamate. In the chronic phase, this contributes to reduced signaling across several neurotransmitter systems, as shown in experimental TBI research (McGuire et al., 2019). Inflammation also reduces the astrocytes’ ability to take up glucose from the bloodstream, limiting the energy supply to neurons (Abbott et al., 2006). Additionally, impaired glutamate uptake combined with a slight increase in astrocyte volume leads to reduced extracellular space and higher extracellular glutamate concentrations. This excess glutamate can diffuse and activate nearby neurons non-specifically (Syková, 2004). Because neuronal activation is an energy-demanding process (Harris et al., 2012), the activation of additional, nonspecific neurons further increases energy consumption at the same time that glucose delivery is compromised. We propose that this combination results in an energy crisis in the brain. This state, which we refer to as BFS, affects multiple neurotransmitter systems and manifests as cognitive, sensory, and emotional symptoms, with profound fatigue as the core feature. Further details of this hypothesis are presented in the article by Johansson and Rönnbäck (Johansson and Rönnbäck, 2025).

In recent years it has been demonstrated that activated or reactive astrocytes shut down calcium-dependent communication between the cells and replace it with extracellular ATP signaling (Rodrigues et al., 2015). ATP and adenosine is well recognized as an extracellular signal substance in inflammatory reactive cells (Shigetomi et al., 2024) and can trigger inflammatory processes that maintain the astrocytes’ dysfunctional state (Guo et al., 2024). Systemic inflammation is recognized as a key driver of persistent fatigue following neurological disease, acquired brain injury, ME/CF, as well as autoimmune diseases (Zielinski et al., 2019; Cervellati et al., 2020; Chaban et al., 2020; Tammelin et al., 2026; Dudova et al., 2025).

### Clinical data

BFS can today not be detected with biological markers or brain imaging of use in the clinic. However, indirectly, altered brain activation and connectivity have been reported to relate to BFS (Skau et al., 2021; Glavå et al., 2025; Skau et al., 2019; Berginström et al., 2018; Wylie et al., 2017).

A systematic review that included neuroimaging studies using functional magnetic resonance imaging (fMRI) and positron emission tomography (PET) across various medical conditions characterized by significant fatigue identified a broad cortical–subcortical network involved in this symptom. The analysis pointed to alterations spanning frontal, limbic, basal ganglia, and parietal regions. Three principal clusters emerged: a frontal–striatal–limbic cluster, a frontal–cingulate cluster, and a parietal cluster, each associated with cognitive, emotional, and somatosensory aspects commonly linked to fatigue. The authors propose converging evidence for a transdiagnostic fatigue network, with key nodes in the lateral frontal cortex, cingulate cortex, insula, thalamus, precuneus, and caudate (Schumann et al., 2025). The BFS with cognitive, sensory and emotional symptoms represents a similar view of fatigue, with symptoms emanating from several brain areas and across different diagnostic groups. However, we hypothesize that the underlying pathology results in a broadly distributed impairment of brain function rather than deficits confined to the regions highlighted above. While some brain networks may be more susceptible than others, a wide range of cognitive and behavioral functions may be affected. Such impairments can have substantial consequences for daily life, influencing occupational performance, social engagement, and quality of life.

## BFS is not depression

BFS and depression represent distinct different clinical states (Johansson et al., 2009; Johansson et al., 2023; Ponsford et al., 2015; Cantor et al., 2008), although depression and fatigue share overlapping symptoms, and an individual may suffer from both depression and BFS. Fatigue after TBI has been suggested to be related to the stroke itself (Ponsford et al., 2015). It did not improve with the antidepressant Fluoxetine following stroke (Choi-Kwon et al., 2007), and uniquely contributes to disability after TBI, even when controlling for injury severity, executive functions, and depression status (Cantor et al., 2008). Brain fatigue is also a recognized as a distinctive state in GD and can occur independently of depression (Johansson et al., 2023).

Differentiating between Brain Fatigue Syndrome (BFS) and depression can be challenging due to overlapping symptoms such as concentration difficulties, reduced initiative, irritability, and sleep disturbances. However, in depression, core symptoms include a persistently depressed mood and diminished interest or pleasure in activities. In contrast, a distinguishing feature of BFS is that individuals retain motivation and interest in activities, but require prolonged recovery. BFS is also characterized by marked fluctuations in symptoms throughout the day and between days. This distinction is crucial when considering treatment. Tailored interventions are required.

## BFS in relation to cognitive function

There is a well-recognized discrepancy between cognitive performance measured with standard neuropsychological tests and how individuals describe their cognitive functioning in everyday life (Johansson et al., 2009). Individuals with BFS often report considerable cognitive difficulties on self-report scales. In research settings, group-level differences in cognitive test can sometimes be detected; however, these differences are typically small and frequently remain within the normal range when assessed in clinical practice (Johansson et al., 2023; Nelson et al., 2021; Föyen et al., 2023). Importantly, cognitive neuropsychological testing is typically conducted in structured, quiet, and predictable environments, which differ markedly from real-life conditions. Everyday life is characterized by constant sensory input, background noise, distractions, and competing demands.

The discrepancy between cognitive tests and self-reported cognitive ability is illustrated in Figure 3 using *Z*-values (a standardized score that converts raw data into a common scale) which allow comparison across cognitive tests and self-reported cognitive difficulties from the MFS, as all values are expressed relative to deviations from the control group (Johansson et al., 2023; Skau et al., 2021; Johansson and Rönnbäck, 2012; Skau et al., 2019). Neuropsychological tests are developed to identify focal brain damage (Lezak et al., 2004) and are not intended to predict function in everyday life (Sbordone, 2010).

**Figure 3:**
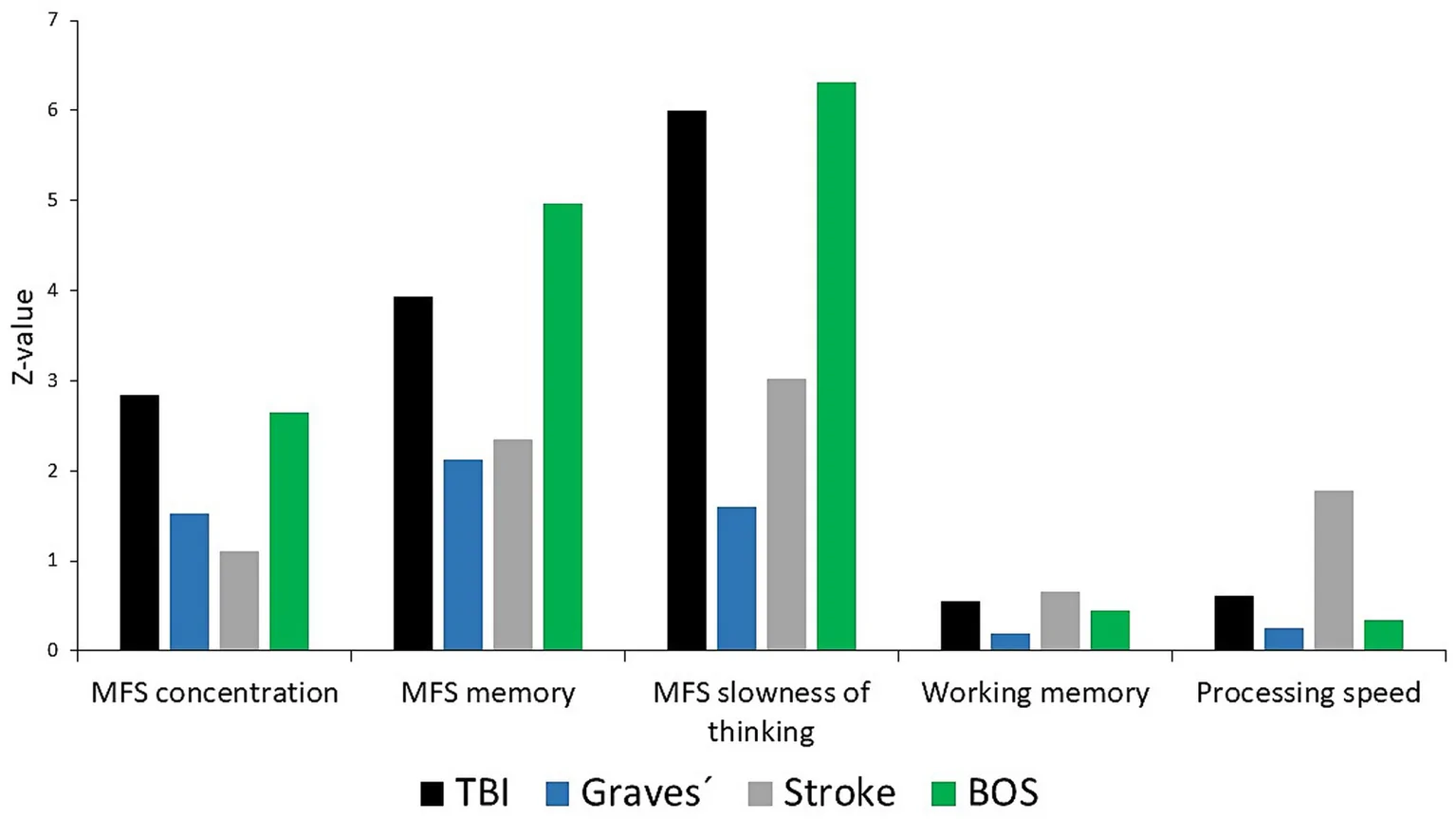
Illustrates the difference in cognitive performance on neuropsychological test from WAIS-IV (processing speed coding and working memory) and how cognitive symptoms (concentration and memory difficulties, slowness of thinking) are reported from Mental Fatigue Scale. Raw scores have been converted to *Z*-values, based on the deviation from non-fatigued controls, demonstrating the discrepancy and the comparability between objective cognitive test outcomes and self‑reported cognitive complaints. Data are from different studies (Johansson et al., 2023; Skau et al., 2021; Johansson and Rönnbäck, 2012; Skau et al., 2019).

The assessment instruments, such as the MFS, are intended to capture aspects of everyday functioning, particularly the capacity to sustain repeated mental activity. We propose that the available cognitive resources are insufficient to meet the demands of real-world situations, despite apparently intact performance under test conditions.

## Treatment

There is currently no effective treatment or medication that cures Brain Fatigue Syndrome (BFS), but within healthcare we can and should offer patients relief based on the knowledge available today. Treatment must also be tailored to the individual’s needs, circumstances, and available energy. Diagnosis-specific symptoms should be treated according to existing recommendations. However, treatment approaches for alleviating BFS may be applicable across different diagnostic groups, and that is what is exemplified here. In this context, we propose and present treatment strategies that may provide relief, including energy-conserving strategies and interventions aimed at alleviating the emotional burden, stress, pain and improve sleep. This is particularly important to address, as for many individuals BFS becomes long-lasting or even chronic.

Diagnosis-specific symptoms should be treated according to existing recommendations.

However, treatment approaches for BFS may be applicable across different diagnostic groups, and that is what is exemplified here.

We are most likely dealing with a reduced energy pool, consistent with the hypothesis presented here. The brain accounts for approximately 20% of the body’s total energy consumption (Mink et al., 1981), and the majority of this energy is used for synaptic transmission (Harris et al., 2012). This means that the brain requires a substantial and continuous energy supply, and when that supply becomes insufficient, difficulties inevitably arise. In short, BFS can be compared to an aging rechargeable battery: the individual becomes “discharged” more quickly and needs extended periods to recover, often repeatedly throughout the day. Furthermore, because the person likely begins the day with already reduced energy levels, indirectly indicated in previous findings (Skau et al., 2019; Berginström et al., 2018; Zhang et al., 2018), they must adapt their daily life to the limited energy available they have already in the morning, as no reserve capacity remains to draw upon. In addition, after effort that requires mental activation and attention, it takes disproportionate long time, sometimes up to several days to regain mental energy. This is particularly important to address, as for many individuals BFS becomes long-lasting or even chronicIn clinical practice, we meet patients with BFS who push themselves far beyond what is healthy, sometimes while still maintaining full-time work. The patients working full-time risk being perceived as healthy, despite struggling significantly, and face an elevated risk of worsening brain fatigue and develop depression and severe exhaustion. Rather than being overlooked, these individuals require preventive support to avoid further deterioration of their health. Clinical experiences indicate that many patients experience setbacks when they increase their working hours, and that fatigue and depression are correlated (Johansson and Rönnbäck, 2014), suggesting an elevated risk of developing depression as BFS worsens.

Healthcare professionals, including physicians, psychologists, occupational therapists, physiotherapists, nurses, and others, play a central role in supporting patients who suffer from BFS. Patient organizations and peer support groups can also provide invaluable assistance.

Following assessment, patients and family members need to be well informed about BFS, its common symptoms, and how these symptoms can influence daily life. Knowledge forms the foundation for developing a person-centered treatment plan. Such a plan should include energy-conserving strategies (Vatwani and Margonis, 2019), such as structure in life concerning, sleep, food and physical activity, taking regular breaks to regain energy (both short and longer breaks), shortening the duration of activities, and planning tasks in advance. Adjustments to both home and work environments are often necessary. An occupational therapist can offer essential guidance in implementing these strategies.

Reduced work ability is frequently reported as a common consequence of BFS (Johansson, 2022; Glise et al., 2010; Fahrenfort et al., 2000; Rutkowski et al., 2021). Temporary or partial disability leave is frequently required, particularly in the early stages of the condition. Successful return to work requires person-centered rehabilitation that integrates daily life and work, with close collaboration between healthcare providers and employers (Vollertsen et al., 2025).

Clinical experience suggests that exceeding one’s energy limits can worsen brain fatigue. It is not possible to increase activity beyond what is sustainable; doing so risks aggravating symptoms. Experience also shows that activity levels must be increased very gradually, both in work situations and in everyday life. This also includes physical activity (Lindegård et al., 2015). The goal is to improve energy levels, reduce symptoms, and manage the condition without delayed reaction to overexertion. Brain fatigue often leads to frustration, anxiety, and sadness when everyday life, and activities that were previously effortless become disrupted, even long after the acute phase has resolved. An additional burden of stress is often added in the form of expectations and demands, both one’s own and those of others. Treatment of stress sensitivity, like all other treatments exemplified here, needs to be adapted to the existing fatigue. Psychological support can be valuable, and a psychologist may provide appropriate and adapted treatment and guidance (Ponsford et al., 2020; Rezaei et al., 2026). When depression or anxiety is present, these conditions should be treated using a combination of medical and psychological interventions tailored to the individual’s needs and taking the brain fatigue into account.

Only a few pharmacological treatments have been studied for reducing fatigue, and we provide examples here. Methylphenidate has been shown to reduce brain fatigue in individuals with TBI, but not improve work capacity and the positive effect on brain fatigue is only seen during ongoing treatment (Johansson et al., 2015), although its effectiveness in other conditions remains unclear. Modafinil has been reported to reduce daytime sleepiness but not fatigue itself, even though some patients may still find it helpful in daily life (Jha et al., 2008; Kaiser et al., 2010). Several other drugs have been investigated, yet all show mixed or inconclusive effects on fatigue (Berginström et al., 2017; Nilsson et al., 2020; Tenovuo et al., 2009). Clinical experience indicates that irritability, emotional instability, or tearfulness can sometimes be alleviated with low-dose antidepressants when needed. Fluoxetine did not reduce fatigue after stroke, although it did improve depressive symptoms (Choi-Kwon et al., 2007). More research is urgently needed, primarily to identify treatments that can directly address fatigue. In the meantime, even partially alleviating medications may be important and should be explored and adapted for different diagnostic groups.

Non-pharmacological group interventions, such as psycho-educative and mindfulness-based therapies for acquired brain injury and multiple sclerosis, have shown promise in reducing fatigue (Glavå et al., 2025; Johansson et al., 2012; Grossman et al., 2010). The Fatigue Management Course, designed to help patients learn strategies tailored to their personal needs may also be helpful (Packer et al., 1995). Cognitive Behavioral Therapy (CBT) can alleviate fatigue when it is adapted for individuals experiencing brain fatigue after TBI (Ponsford et al., 2020; Nguyen et al., 2017). A review reported that CBT is effective for fatigue in rheumatoid arthritis, neurological disorders, and post-infectious conditions. In addition, it suggested that similar fatigue treatment methods can be applied across different disorders (Steen et al., 2025). From a meta-analysis, CBT can be supportive for chronic fatigue syndrome (Kolala et al., 2025). Blue light therapy shows mixed results. It may reduce daytime sleepiness, and it has been suggested that improving circadian regulation could help alleviate fatigue after TBI (Connolly et al., 2021; Sinclair et al., 2014). Neurophysiological therapeutic techniques such as transcranial direct current stimulation (tDCS) and transcranial magnetic stimulation (TMS) have also been investigated, but findings regarding their effects on fatigue remain variable (Charvet et al., 2025; Dong et al., 2021; Gaede et al., 2017; Kamamuta et al., 2023). As with medications, only a limited number of treatment studies exist, underscoring the need for further research in this area.

Physical activity is important for overall wellbeing but must be carefully tailored to each individual’s capacity and level of brain fatigue. Support from a physiotherapist can be highly beneficial. One study combining graded activation with cognitive therapy demonstrated a slight reduction in fatigue after stroke, although no significant difference was found between graded activation and cognitive therapy alone (Zedlitz et al., 2012). Physical activity has been identified as a promising intervention for reducing fatigue in progressive neurological disorders, particularly MS (Röttgering et al., 2026). A review indicates that the effects of physical activity on chronic fatigue are uncertain, including concerns regarding the risk of side effects from exercise therapy (Larun et al., 2024).

In summary, all therapeutic approaches must be adapted to the patient’s fatigue and personal needs. This does not imply that patients are unable to participate in therapy or activities, but rather that longer treatment periods, a slower pace and repetitions may be required.

## Conclusion

BFS appears to be a cross-diagnostic phenomenon affecting a substantial proportion of individuals and is often poorly recognized, despite its profound and debilitating impact on everyday life and seemingly intact performance under test conditions. We suggest that BFS represents a manifestation of generalized cerebral involvement; however, individuals may concurrently exhibit disease-specific symptoms or deficits related to a brain injury. Symptoms associated with BFS typically persist for the duration of the underlying brain-affecting condition or until recovery occurs. Nevertheless, in a subset of individuals, BFS persists beyond the expected recovery period. Chronic BFS may adversely affect occupational functioning and social participation. At present, the determinants of vulnerability to persistent BFS remain unclear and curative treatments are currently lacking. However, patients can be supported through interventions aimed at alleviating fatigue and reducing its impact on daily functioning. A core responsibility of healthcare services is to alleviate suffering, provide adequate support, and offer individualized management strategies. There is a clear need for intensified research into the pathophysiology of BFS, as well as the development of specific diagnostic tools and effective treatment options.

## Data Availability

The raw data supporting the conclusions of this article will be made available by the authors, without undue reservation.
